# An original Eurasian haplotype, HLA-DRB1*14:54-DQB1*05:03, influences the susceptibility to idiopathic achalasia

**DOI:** 10.1371/journal.pone.0201676

**Published:** 2018-08-09

**Authors:** Janette Furuzawa-Carballeda, Joaquín Zuñiga, Diana I. Hernández-Zaragoza, Rodrigo Barquera, Eduardo Marques-García, Luis Jiménez-Alvarez, Alfredo Cruz-Lagunas, Gustavo Ramírez, Nora E. Regino, Ramón Espinosa-Soto, Edmond J. Yunis, Fernanda Romero-Hernández, Daniel Azamar-Llamas, Enrique Coss-Adame, Miguel A. Valdovinos, Samuel Torres-Landa, Axel Palacios-Ramírez, Blanca Breña, Edgar Alejandro-Medrano, Axel Hernández-Ávila, Julio Granados, Gonzalo Torres-Villalobos

**Affiliations:** 1 Department of Immunology and Rheumatology, Instituto Nacional de Ciencias Médicas y Nutrición Salvador Zubirán, Mexico City, Mexico; 2 Laboratory of Immunobiology and Genetics, Instituto Nacional de Enfermedades Respiratorias Ismael Cosío Villegas, Mexico City, Mexico; 3 School of Medicine and Health Sciences, Instituto Tecnológico y de Estudios Superiores de Monterrey, Mexico City, Mexico; 4 Department of Archaeogenetics, Max Planck Institute for the Science of Human History, Jena, Germany; 5 Department of Cancer Immunology and AIDS, Dana Farber Cancer Institute, Harvard Medical School, Boston, MA, United States of America; 6 Department of Experimental Surgery, Instituto Nacional de Ciencias Médicas y Nutrición Salvador Zubirán, Mexico City, Mexico; 7 Department of Gastroenterology, Instituto Nacional de Ciencias Médicas y Nutrición Salvador Zubirán, Mexico City, Mexico; 8 Department of Transplants, Instituto Nacional de Ciencias Médicas y Nutrición Salvador Zubirán, Mexico City, Mexico; 9 Department of Surgery, Instituto Nacional de Ciencias Médicas y Nutrición Salvador Zubirán, Mexico City, Mexico; University of Texas Health Science Center at San Antonio, UNITED STATES

## Abstract

Idiopathic achalasia is a relatively infrequent esophageal motor disorder for which major histocompatibility complex (MHC) genes are well-identified risk factors. However, no information about HLA-achalasia susceptibility in Mexicans has previously been reported. We studied a group of 91 patients diagnosed with achalasia and 234 healthy controls with Mexican admixed ancestry. HLA alleles and conserved extended haplotypes were analyzed using high-resolution HLA typing based on Sanger and next-generation sequencing technologies. Admixture estimates were determined using HLA-B and short tandem repeats. Results were analyzed by non-parametric statistical analysis and Bonferroni correction. P-values < 0.05 were considered significant. Patients with achalasia had 56.7% Native American genes, 24.7% European genes, 16.5% African genes and 2.0% Asian genes, which was comparable with the estimates in the controls. Significant increases in the frequencies of alleles DRB1*14:54 and DQB1*05:03 and the extended haplotypes DRB1*14:54-DQB1*05:03 and DRB1*11:01-DQB1*03:01, even after Bonferroni correction (*p*C<0.05), were found in the achalasia group compared to those in the controls. Concluding, the HLA class II alleles HLA-DRB1*14:54:01 and DQB1*05:03:01 and the extended haplotype are risk factors for achalasia in mixed-ancestry Mexican individuals. These results also suggest that the HLA-DRB1*14:54-DQB1*05:03 haplotype was introduced by admixture with European and/or Asian populations.

## Introduction

Idiopathic achalasia is a relatively uncommon major motility disorder of the esophagus that inflicts substantial morbidity upon affected individuals [1]. Clinically, idiopathic achalasia is characterized by dysphagia with aperistalsis and the absence of lower esophageal sphincter (LES) relaxation [2]. While the factors that influence achalasia development remain to be completely elucidated, viral, autoimmune and genetic host factor influences have been studied [3]. In this regard, familial aggregation in twin studies has suggested that genetic factors play an important role in the pathogenesis of this condition [4]. The contributions of major histocompatibility complex (MHC) class II loci in achalasia were first explored by Wong et al. in 1989 [5], showing an association of DQw1 with higher susceptibility to the disease. In 1998, De la Concha and colleagues found a significant association between the allele HLA-DQA1*01:01 and a protective role of HLA-DQB1*02 in a small group of Caucasian European patients [6]. Other studies have described a protective effect of the conserved extended haplotype (CEH) DRB1*15:01-DQA1*01:02-DQB1*06:02 [7]. Some association studies in large cohorts of achalasia patients with central European ancestry have demonstrated that haplotypes bearing HLA-DQB1*05:03 and DQB1*06:01 are linked with susceptibility to achalasia [8]. Additionally, differences in the distribution of these achalasia-risk HLA class II alleles among Central Europeans appear to influence the prevalence of the disease in Europe [9].

The frequency of CEHs and specific block combinations of HLA genes varies between major ethnic groups and/or different continental ancestries, and these variations can be used as measurements of MHC genetic diversity in autoimmune conditions [10,11].

Various population genetics studies have revealed that Mexican mixed-ancestry populations have complex genetic structures with contributions from Native American (50–60%), European (25–40%), African (4–12%), and more recently, Asian (1%) biological roots [11–14]. In this context, the role of HLA and ethnic backgrounds in the susceptibility of Mexicans to achalasia has not been explored, and we believe that the identification of specific HLA haplotypes associated with achalasia in Mexicans will be helpful for understanding the genetic background related to this condition. Thus, this study aimed to describe the distribution of HLA class I and class II blocks and CEHs and their most likely ancestral origins using high-resolution HLA typing in a group of Mexican mixed-ancestry patients with achalasia.

## Materials and methods

### Patient samples

Eligible patients included those born in Mexico whose parents and grandparents were also born in Mexico, with a diagnosis of idiopathic achalasia as described below. The diagnosis of achalasia was based on clinical evaluations as well as on esophagram, high-resolution manometry (classified based on Chicago v3.0) [15] and endoscopy results. All patients were recruited between 2014 and 2017 from the Outpatient Clinics of Gastroenterology and Surgery of the National Institute for Medical Sciences and Nutrition Salvador Zubirán in Mexico City, which is a referral center for this condition. We excluded patients from study participation according to diagnosis of secondary achalasia due to Chagas disease, esophageal stricture, gastric, esophageal cancer or esophageal scleroderma. A total of 182 HLA class I and class II haplotypes from 91 patients diagnosed with achalasia were analyzed in this study. All achalasia patients were of Mexican ancestry, and admixture estimations using HLA markers revealed a greater proportion of Native American genetic contributions, followed by an important component of European alleles.

As the control group, 234 unrelated Mexican admixed individuals were studied, including a group of 40 Mexican admixed families, providing a total of 468 haplotypes for this HLA-disease association study. All participants had Mexican ancestry, and their parents and grandparents were born in Mexico. Admixture estimations using *HLA-B* and short tandem repeats (STRs) were performed in this group of controls to determine whether their genetic backgrounds was comparable to that of achalasia patients.

### Ethics statement

The Institutional Review Board of the National Institute for Medical Sciences and Nutrition Salvador Zubirán (INCMNSZ) and the National Institute for Respiratory Diseases (INER) reviewed and approved the protocols for genetic studies. All subjects provided written informed consent for these studies, and they authorized the storage of their DNA samples at INER or INCMNSZ repositories for this and future studies. In this study, we collected samples only from adults older than 18 years.

### Sanger sequencing-based HLA typing

Genomic DNA was obtained from peripheral blood mononuclear cells (PBMCs) using the QIAamp DNA mini kit (*Qiagen*, *Valencia*, *CA*, *USA*). High-resolution HLA class I and class II typing was performed using a sequence-based method (SBM) as described previously [11,16,17]. Briefly, we amplified exons 2 and 3 from *HLA-A*, *HLA-B* and *HLA-C* and exon 2 from *HLA-DRB1* and *HLA-DQB1*. Polymerase chain reactions (PCRs) utilized 1.5 mm KCl, 1.5 mM MgCl2, 10 mM Tris-HCl (pH 8.3), 200 mM dNTPs, 10 pM of each primer, 30 ng of DNA and 0.5 U of *Taq* DNA polymerase in a final volume of 25 μl. Amplifications were performed on a PE9700 thermal cycler (*Applied Biosystems*, *Foster City*, *CA*, *USA*) under the following cycling conditions: 95°C for 30 s, 65°C for 30 s, 72°C for 1 min, preceded by 5 min at 95°C and followed by a final elongation step at 72°C for 5 min. The amplified products were sequenced independently in both directions using BigDye TerminatorTM chemistry on the ABI PRISM® 3730xl Genetic Analyzer (*Applied Biosystems*). Data were analyzed with match tools allele assignment software (*Applied Biosystems*) using the IMGT/HLA sequence database alignment tool (http://www.ebi.ac.uk/imgt/hla/align.html). Ambiguities were solved using group-specific sequencing primers (GSSPs) that had been previously reported and validated [16,17].

### High-resolution HLA typing by next-generation sequencing

We also used the next-generation sequencing Trusight Illumina (*Illumina*, *San Diego*, *CA*, *USA*) HLA system to confirm HLA allele-level typing. Briefly, genomic DNA samples from achalasia patients were adjusted to a working concentration of 10 ng/μL using the real-time PCR assay Quibit BR and Qubit equipment (*Thermo Fisher Scientific*, *Waltham*, *MA*, *USA*). Long-range PCR templates of *HLA-A*, *HLA-B*, *HLA-C*, *HLA-DRB1* and *HLA-DQB1* loci were prepared using specific primers included in the Trusight HLA Pre 24 sample kit (*Illumina*) and MasterAmpTM Extra-Long DNA Polymerase (*Lucien Corporation*, *Middleton*, *WI*, *USA*). PCR reactions were performed in a 96-well plate on the 9700 PE thermal cycler (*Applied Biosystems/Thermo Fisher Scientific*) under the following PCR conditions: 25 μl of HPM (HLA-PCR Mix), 2 μl of MasterAmpTM Extra-Long DNA Polymerase, 13 μl of water and 5 μl of gDNA (10 ng/μl). For HLA-DQB1, the locus conditions were 94°C for 3 min; followed by 10 cycles at 94°C for 30 sec, 55°C for min, 72°C for 15 min; 20 cycles of 94°C for 30 sec, 60°C for 2 min, 72°C for 15 min, 72°C for 10 min; and a final hold at 10°C. Simultaneously, the PCR reactions for HLA-A, HLA-B, HLA-C and DRB1 loci were performed under the following conditions: initial denaturation at 94°C for 3 min, 30 cycles at 94°C for 30 sec, 60°C for 2 min, 68°C for 15 min, 68°C for 10 min and a final hold at 10ºC. PCR products were confirmed by 1% agarose gel electrophoresis. Using magnetic beads (LNA1, LNB1, Trusight HLA, *Illumina*), we proceeded to normalize the concentrations of the PCR products of all loci for multiplex library preparation and next-generation sequencing. After normalization, 40 μl of each PCR product was used for fragmentation (800 and 1200 pb), and fragmentation buffers HTM and HTB (Trusight HLA Pre-PCR 24, *Illumina*) were added to the reaction (10 μl each) and incubated at 58°C for 12 min in the presence of sequencing primers. The purified fragmented PCR products were pooled, and adaptor addition was performed using the Nextera XT DNA sample preparation kit (*Illumina*). Amplification was performed under the following conditions: denaturation at 72°C for 3 min and 98°C for 30 sec, followed by 10 cycles at 98°C for 10 sec, 60°C for 30 sec, 72°C for 5 min, and a final hold at 10°C. Seven microliters of the PCR sequencing products were denatured with 10 μl of 0.1N NaOH and sequenced on a MiSeq instrument using the paired-end 300 cycle (2 x 150 bp paired-end) MiSeq Reagent Kit (*Illumina*).

### Next-generation sequencing data analysis

After the sequencing, MiSeq Reporter analysis software generated FASTQ sequence files, BAM alignment files and allele calling were generated using Trusigh HLA ASSIGN 2.1 software (v2.1.0.943RUO.msi, *Illumina*). The software used reference sequences from the IMGT/HLA database (release 3.28.0).

### Assignation of HLA class I and class II conserved extended haplotypes (CEHs)

Allele, haplotype and CEH HLA class I and class II frequencies at allelic resolution from both achalasia patients and controls were obtained by family segregation analysis. Maximum likelihood haplotype frequencies for two-point, three-point, four-point and five-point associations were estimated using an expectation-maximization (EM) algorithm provided by the computer program *Arlequin* ver. 3.1 [18]. Hardy-Weinberg equilibrium (HWE) at a locus-by-locus level was also calculated using this software. The polymorphism information content (PIC), observed heterozygosity (OH), and expected heterozygosity (EH) for each HLA class I and class II locus were also calculated. CEHs of known African, Asian and Caucasian origin were assigned based on previous reported frequencies in different ethnic groups including Mexican admixed and Native American populations [11]. Delta (Δ) and relative delta (Δ’) values were calculated using previously described standardized methods to measure linkage disequilibrium (LD) [19], defined as the non-random association of alleles at two or more loci and their statistical significance <0.05.

### Admixture estimations using HLA genes

Admixture estimations in achalasia patients and healthy controls were obtained by the maximum likelihood method using the population genetics *Leadmix* software [20] with *k* = 4 parental populations (Europe, Africa, Asia, and America) and *HLA-B* as the genetic estimator. European components were estimated based on HLA data from southern Portugal and USA inhabitants [21,22]. African components were calculated using parental populations from Nandi from Kenia [23], and Native American Mexican components were calculated using previously reported HLA data from Oaxaca Mixtecs, a population from southeastern Mexico [24], and Tarahumaras from Chihuahua in northern Mexico [25]. Finally, Southern Han Chinese data (N = 281) were used to mate the Asian contributions in both samples [26].

### Admixture estimations using STRs

To estimate the genetic backgrounds of the healthy controls, we used the distribution of autosomal STR markers (*CSF1PO*, *FGA*, *THO1*, *TPOX*, *VWA*, *D3S11358*, *D5S818*, *D7S820*, *D8S1179*, *D13S317*, *D16S539*, *D18S51*, *D21S11*, *D19S433*, and *D2S1338*) along with amelogenin using the Applied Biosystems AmpF/STR Identifier Kit (*Applied Biosystems*). PCR amplification and capillary electrophoresis were carried out as previously described [14]. An analysis of admixture estimation using STR data was performed using a model-based clustering method with *Structure* software v. 2.3.4 [27]. For this analysis, we assumed that *k* = 3 for the parental populations and performed 10,000 dememorization steps using STR data previously published in Spaniards [28], Fang Africans [29], and Native American Huastecos [30] and Tepehuas [31] populations from the central region of Mexico.

### Antinuclear antibodies (ANAs) testing

Only patients in the idiopathic achalasia group newly diagnosed and without previous treatment donated blood samples, which were used for ANAs assessment by the indirect immunofluorescence of HEp-2 cells with the IgG isotype (*Inova Diagnostics Inc*., *San Diego*, *CA*, *USA*). Positivity was assigned according to our local cut-off values (*i*.*e*., speckled: > 1:160; nucleolar: > 1:40; cytoplasmic: > 1:40; mitochondrial: > 1:160; others: > 1:40) [32].

### Statistical analysis

Differences in the frequencies of HLA class I and class II alleles as well as in HLA conserved haplotypes were analyzed using *X*^2^ or Fisher’s exact test, and *p*-values less than 0.05 were considered statistically significant. If appropriate, the p-values were also corrected using the Bonferroni (for allele frequencies, multiplying the original *p*-value by the number of alleles) or Yates methods (for block and haplotype frequencies); odds ratios and 95% confidence intervals (CI) were calculated to measure association strength (EPIINFO v7 software).

Numerical variables were analyzed using Student’s *t*-test, and Pearson correlations were also calculated using *SPSS* software, version 15 (*IBM Corp*., *Armonk*, *NY*, *USA*).

The minimal data set necessary to replicate the study findings is in the Supporting Information files (S1 Data).

## Results

### Clinical and demographic characteristics

The clinical and demographic characteristics of the Mexican patients with achalasia are summarized in Table 1. The patients (66% female) had a mean age of 42.3 ± 15.8 years, while the mean age of the control patients (51% female) was 38.0 ± 15.0 years. As expected, 100% of the patients diagnosed with achalasia had dysphagia, 91% had regurgitation, 69% heartburn and 88% exhibited an important weight loss. The mean time of disease onset was 24.3 months, and the percentage of autoimmune disease among the achalasia patients was 13%. The most frequent associated disease was thyroid autoimmune disease (6.3%). Two patients (2.2%) had more than one concomitant autoimmune diseases (1 with both hyperthyroidism and vitiligo and 1 with hypothyroidism, scleroderma and rheumatoid arthritis), 1 patient (1.1%) had Sjögren’s syndrome, 1 patient (1.1%) had Guillain-Barré syndrome, and 1 patient (1.1%) had ankylosing spondylitis, as previously described by Romero-Hernández et al. [33] More than 25% of the patients had been exposed to wood smoke, and 33% had been exposed to tobacco smoke.

**Table 1:** Clinical and demographic information of Mexican patients with achalasia.

Variable	Achalasia (n = 91)	Type I Achalasia (n = 15)	Type II Achalasia (n = 71)	Type III Achalasia (n = 5)
Age, years, mean±SD	42.3±15.8	45.3±17.6	41.1±14.9	48.7±17.8
Female, n (%)	66 (73)	9 (60)	53 (75)	4 (80)
Male, n (%)	25 (27)	6 (40)	18 (25)	1 (20)
Dysphagia, n (%)	91 (100)	15 (100)	71 (100)	5 (100)
Regurgitation, n (%)	83 (91)	13 (87)	66 (93)	4 (80)
Heartburn, n (%)	63 (69)	5 (33)	56 (79)	2 (40)
Weight loss, n (%)	80 (88)	11 (73)	64 (90)	5 (100)
Time of disease diagnosis, months mean±SD	24.3±35.1	38.0±37.5	21.8±26.9	11.6±7.8
Autoimmune disease, n (%)	12 (13)	2 (13)	8 (11)	2 (40)
ANA autoantibodies, n (%)	62 (68)	9 (60)	49 (69)	4 (80)
Wood smoke, n (%)	24 (25)	4 (27)	18 (25)	2 (40)
Tobacco use, n (%)	30 (33)	5 (33)	19 (27)	3 (60)

ANAs testing was performed on all patients with achalasia, showing positivity in 62 (68%). The most prevalent pattern observed was speckled, followed by nucleolar and homogenous.

### HLA genetic diversity and admixture estimations revealed no differences in the proportions of Native American or European components between Mexican admixed patients with achalasia and healthy controls

Maximum likelihood analysis revealed that the *HLA-DQB1* locus in patients with achalasia and the *HLA-DRB1* locus in controls had marginal significant deviations from Hardy-Weinberg equilibrium (HWE) after Bonferroni correction (*p* <0.05). The degrees of HLA class I and class II locus polymorphisms in both the Mexican mixed-ancestry patients with achalasia and the controls were analyzed using PIC values >0.5. As expected, the *HLA-B* and *HLA-DRB1* loci were the most polymorphic in both groups, whereas *HLA-DQB1* was the least polymorphic locus. The power of discrimination (PD) values of each locus ranged from 0.9538 to 0.9856 in achalasia patients and from 0.9447 to 0.9907 in the controls (Table 2).

**Table 2:** Estimations of genetic diversity of HLA class I and class II loci in Mexican admixed individuals with achalasia and controls.

	Achalasia					Controls				
HLA loci	O.H.	E.H.	pCorr	PIC	PD	O.H.	E.H.	pCorr	PIC	PD
HLA-A	0.8557	0.8918	ns	0.8794	0.9740	0.8718	0.8919	ns	0.8776	0.9761
HLA-B	0.9588	0.9698	ns	0.9623	0.9856	0.9487	0.9668	ns	0.9544	0.9907
HLA-C	0.8454	0.9012	ns	0.8891	0.9723	0.9009	0.8947	ns	0.8845	0.9767
HLA-DRB1	0.8969	0.9265	ns	0.9171	0.9793	0.9013	0.9193	0.03	0.9123	0.9835
HLA-DQB1	0.7835	0.8555	0.0100	0.8354	0.9538	0.8205	0.8256	ns	0.8020	0.9447

**pCorr**: p corrected value after Bonferroni correction, **O.H.:** Observed heterozygosity, **E.H.:** Expected heterozygosity, **PIC:** polymorphism information contents. **PD:** Power of discrimination

Admixture estimations using *HLA-B* as the genetic estimator revealed that among the patients with achalasia, 56.7% of their genes were of Native American descent, 24.7% were of European descent, 16.5% were of African descent and 2.0% were of Asian descent. In the healthy controls group, 59.8% Native American, 25.7% European, 14.1% African and 1.8% Asian contributions were found. These results were comparable with the estimates in the controls obtained using other polymorphic markers, such as STRs (Native American contribution: 60.5%, European: 25.9%, and African: 13.6%). These findings demonstrate that the achalasia patients and the controls had comparable genetic backgrounds in the context of their parental populations.

### Achalasia is associated with HLA class II alleles in Mexicans

The frequencies of HLA class I (*HLA-A*, *HLA-B*, *HLA-C*) alleles are summarized in S1, S2 and S3 Tables, respectively. The most frequent *HLA-A* alleles in both the achalasia and control groups were A*02:01, A*24:02 and A*02:06, with frequencies greater than 10%. For the *HLA-B* locus, the most common alleles in achalasia patients were B*39:05, B*35:01 and B*44:03, with gene frequencies of 0.0989, 0.0549 and 0.0495, respectively. In this regard, the most common *HLA-B* alleles in the controls were B*39:05, B*39:06 and B*51:01, with allele frequencies of 0.0791, 0.0684 and 0.0598, respectively. Regarding the *HLA-C* locus, the most common alleles in the achalasia patients were C*07:02, C*04:01 and C*12:03, whereas in the controls, the most common alleles were C*07:02, C*04:01 and C*01:02. No significant differences in the distribution of HLA class I alleles among patients with achalasia and the controls were observed.

We found a significant increase in the frequency of the DRB1*14:54 allele in the achalasia group (G.F. = 0.0604) compared to that in the control group (G.F. = 0.0171, *pC* = 0.0200, OR = 3.7, 95% CI = 1.35–10.26). We also found a significant increase in the frequency of the HLA-DRB1*0407 allele (*pC* = 0.01, OR = 1.85; 95% CI = 1.14–3.01) in the achalasia group. In contrast, a decreased frequency of the DRB1*08:02 allele was observed (Table 3).

**Table 3:** Gene frequencies of HLA-DRB1 in achalasia patients and healthy controls.

	Achalasia (N = 182)		Controls (N = 468)			
Allele	n	G.F.	n	G.F.	*pCorr*	OR (95%CI)
DRB1*01:01	5	0.0274	9	0.0192	ns	
DRB1*01:02	5	0.0274	11	0.0235	ns	
DRB1*01:03	1	0.0054	3	0.0064	ns	
DRB1*03:01	7	0.0385	15	0.0321	ns	
DRB1*04:01	3	0.0164	3	0.0064	ns	
DRB1*04:02	2	0.0109	10	0.0214	ns	
DRB1*04:03	1	0.0054	10	0.0214	ns	
DRB1*04:04	8	0.0439	31	0.0662	ns	
DRB1*04:05	1	0.0054	1	0.0021	ns	
DRB1*04:07	**36**	**0.1978**	**55**	**0.1175**	**0.0114**	**1.85 (1.14–3.01)**
DRB1*04:08	1	0.0054	1	0.0021	ns	
DRB1*04:11	3	0.0164	9	0.0192	ns	
DRB1*07:01	12	0.0659	33	0.0705	ns	
DRB1*08:01	1	0.0054	1	0.0021	ns	
DRB1*08:02	**22**	**0.1208**	**91**	**0.1944**	**0.0284**	**0.57 (0.33–0.96)**
DRB1*10:01	3	0.0164	6	0.0128	ns	
DRB1*11:01	6	0.0329	6	0.0128	ns	
DRB1*11:04	1	0.0054	8	0.0171	ns	
DRB1*12:01	2	0.0109	2	0.0043	ns	
DRB1*13:01	5	0.0274	12	0.0256	ns	
DRB1*13:02	9	0.0495	10	0.0214	ns	
DRB1*13:03	1	0.0054	3	0.0064	ns	
DRB1*13:05	1	0.0054	1	0.0021	ns	
DRB1*14:02	3	0.0164	11	0.0235	ns	
DRB1*14:06	12	0.0659	47	0.1004	ns	
DRB1*14:54	**11**	**0.0604**	**8**	**0.0171**	**0.0200**	**3.7 (1.35–10.26)**
DRB1*15:01	5	0.0274	17	0.0363	ns	
DRB1*15:02	3	0.0164	5	0.0107	ns	
DRB1*15:03	2	0.0109	1	0.0021	ns	
DRB1*16:02	10	0.0549	30	0.0641	ns	

**G.F.:** Gene Frequency; **ns:** not significant; ***pCorr*:** *p* Corrected value using Bonferroni method; **OR:** Odds ratio; **95%CI:** 95% Confidence Interval.

Analysis of *HLA-DQB1* alleles revealed a significant association between the DQB1*05:03 allele and achalasia (*pC* value = 0.0036, OR = 4.06; 95% CI = 1.52–11.07, Table 4).

**Table 4:** Gene frequencies of HLA-DQB1 in achalasia patients and healthy controls.

	Achalasia (N = 182)		Controls (N = 468)			
Allele	n	G.F.	n	G.F.	*pCorr*	OR (95%CI)
DQB1*02:01	7	0.0385	15	0.0321	ns	
DQB1*02:02	11	0.0604	28	0.0598	ns	
DQB1*03:01	36	0.1978	116	0.2479	ns	
DQB1*03:02	48	0.2637	115	0.2457	ns	
DQB1*03:03	1	0.0054	10	0.0214	ns	
DQB1*03:04	2	0.0109	ND			
DQB1*04:02	26	0.1429	96	0.2051	ns	
DQB1*05:01	15	0.0824	32	0.0684	ns	
DQB1*05:03	**12**	**0.0659**	**8**	**0.0171**	**0.0036**	**4.06 (1.52–11.07)**
DQB1*06:01	3	0.0164	5	0.0107	ns	
DQB1*06:02	7	0.0384	17	0.0363	ns	
DQB1*06:03	5	0.0274	7	0.0150	ns	
DQB1*06:04	7	0.0384	10	0.0214	ns	
DQB1*06:09	2	0.0109	ND			

**G.F.:** Gene Frequency; **ns:** not significant; ***pCorr*:** *p* Corrected value using Bonferroni method; **OR:** Odds ratio; **95%CI:** 95% Confidence Interval.

### Distribution of the HLA-C/B and DRB1/DQB1 blocks confirm the relevance of the HLA class II region in the susceptibility of admixed Mexicans to achalasia

In this study, we classified HLA class I (*HLA-C*/*-B*) and class II (*HLA-DRB1*/*-DQB1*) blocks according to their most probable ancestry (MPA) in both the achalasia group and the control group. No significant differences in the distributions of the *HLA-C/-B* blocks from Native American, European, Asian and African MPAs were detected between the patients and controls (S4 Table). In the achalasia patients, 7 Native American-specific *HLA-DRB1*/*-DQB1* haplotypes, 21 European haplotypes, two African MPA haplotypes, two Asian MPA haplotypes and five haplotypes of unknown origin were detected. Analysis of the *HLA-DRB1/-DQB1* blocks revealed an association between Eurasian origin haplotypes and achalasia (Table 5). The haplotype HLA-DRB1*11:01/-DQB1*03:01 was markedly more common in the achalasia group (*pC* value = 0.008, OR = 7.94: 95% CI = 1.65–57.29). We also found a significant increase in the frequency of the haplotype HLA-DRB1*14:54/-DQB1*05:03 in patients with achalasia compared to that in the controls (*pC* = 0.009, OR = 4.06; 95% CI = 1.57–11.09, Table 5).

**Table 5:** Frequencies of HLA-DRB1/-DQB1 block in achalasia patients and healthy controls.

		Achalasia (N = 182)			Controls (N = 468)				
	*HLA-DRB1*/*-DQB1* haplotypes	n	H.F.	Δ'	n	H.F.	Δ'	*pCorr*	OR (95%CI)
**Amerindian**	DRB1*04:07-DQB1*03:02	32	0.1758	0.9628	53	0.1133	0.9518	ns	
	DRB1*08:02-DQB1*04:02	23	0.1263	1.0000	89	0.1902	0.9723	ns	
	DRB1*14:06-DQB1*03:01	15	0.0824	1.0000	46	0.0983	0.9717	ns	
	DRB1*16:02-DQB1*03:01	10	0.0549	1.0000	30	0.0641	1.0000	ns	
	DRB1*14:02-DQB1*03:01	2	0.0109	0.5801	11	0.0235	1.0000	ns	
	DRB1*04:11-DQB1*03:02	1	0.0054	0.1081	8	0.0171	0.8526	ns	
	DRB1*04:11-DQB1*04:02	1	0.0054	0.2161	1	0.0022	-0.4595	ns	
**European**	DRB1*03:01-DQB1*02:01	7	0.0384	1.0000	15	0.0320	1.0000	ns	
	DRB1*11:01-DQB1*03:01	**6**	**0.0329**	**1.0000**	**2**	**0.0043**	**0.1130**	**0.008****ª**	**7.94 (1.65–57.29)**
	DRB1*13:01-DQB1*06:03	5	0.0274	1.0000	6	0.0128	1.0000	ns	
	DRB1*15:01-DQB1*06:02	5	0.0274	1.0000	15	0.0320	0.8779	ns	
	DRB1*04:02-DQB1*03:02	2	0.0109	1.0000	10	0.0214	1.0000	ns	
	DRB1*04:01-DQB1*03:02	2	0.0109	0.5540	3	0.0064	1.0000	ns	
	DRB1*11:04-DQB1*03:01	1	0.0054	1.0000	8	0.0171	1.0000	ns	
	DRB1*07:01-DQB1*03:03	1	0.0054	1.0000	5	0.0107	0.462	ns	
**European shared with other populations**	DRB1*14:54-DQB1*05:03	**12**	**0.0659**	**1.0000**	**8**	**0.0171**	**1.0000**	**0.009****º**	**4.06 (1.57–11.09)**
	DRB1*07:01-DQB1*02:02	11	0.0604	1.0000	28	0.0598	1.0000	ns	
	DRB1*13:02-DQB1*06:04	7	0.0384	1.0000	9	0.0192	0.8978	ns	
	DRB1*04:04-DQB1*03:02	6	0.0329	0.6655	29	0.0620	0.9144	ns	
	DRB1*01:01-DQB1*05:01	5	0.0274	1.0000	9	0.0192	1.0000	ns	
	DRB1*01:02-DQB1*05:01	5	0.0274	1.0000	11	0.0235	1.0000	ns	
	DRB1*15:02-DQB1*06:01	3	0.0164	1.0000	5	0.0107	1.0000	ns	
	DRB1*01:03-DQB1*05:01	1	0.0054	1.0000	3	0.0064	1.0000	ns	
	DRB1*04:03-DQB1*03:02	1	0.0054	1.0000	10	0.0214	1.0000	ns	
	DRB1*04:05-DQB1*03:02	1	0.0054	1.0000	1	0.0022	1.0000	ns	
	DRB1*08:01-DQB1*04:02	1	0.0054	1.0000	1	0.0022	1.0000	ns	
	DRB1*13:03-DQB1*03:01	1	0.0054	1.0000	3	0.0064	1.0000	ns	
	DRB1*13:05-DQB1*03:01	1	0.0054	1.0000	1	0.0022	1.0000	ns	
**African**	DRB1*10:01-DQB1*05:01	3	0.0164	1.0000	5	0.0107	0.8211	ns	
	DRB1*15:03-DQB1*06:02	2	0.0109	1.0000	1	0.0022	1.0000	ns	
**Asian**	DRB1*04:04-DQB1*04:02	2	0.0109	0.1182	2	0.0043	-0.6862	ns	
	DRB1*12:01-DQB1*03:01	2	0.0109	1.0000	1	0.0022	0.3348	ns	
**Unknown**	DRB1*04:01-DQB1*03:01	1	0.0054	0.1601	ND				
	DRB1*04:07-DQB1*05:03	1	0.0054	-0.6151	ND				
	DRB1*04:08-DQB1*03:04	1	0.0054	1.0000	ND				
	DRB1*04:11-DQB1*05:01	1	0.0054	0.2774	ND				
	DRB1*14:02-DQB1*03:04	1	0.0054	0.4921	ND				

**ª**Uncorrected *p* value: 0.001;

**º** Uncorrected *p* value: 0.0008;

**H.F.:** Haplotype Frequency; **ns:** not significant; **ND:** Not detected; **∆'**: Delta max; ***p*Corr:** *p* Corrected value using Bonferroni method; **OR:** Odds ratio; **95%CI:** 95% Confidence Interval.

We also found a significant correlation between the presence of HLA-DRB1*14:54-carrying haplotypes in patients with achalasia that use tobacco (*p* = 0.02) and patients with BMIs higher than 25 (*p* = 0.03). Other significant correlations with other clinical variables and the HLA-DQB1*06:03 allele was not detected. Next-generation HLA sequencing allowed to us to determine that the DRB1*14:54-DQB1*05:03-carrying haplotypes in achalasia patients were DRB1*14:54:01-DQA1*01:04:01-DQB1*05:03:01-DPA1*01:03:01.

### Analysis of HLA class I/class II CEHs in achalasia patients and controls

The distributions of HLA class I/class II CEHs and their MPAs in achalasia patients and controls are summarized in Table 6, and this analysis was extended to *HLA-A* in Table 7. Interestingly, unlike in the HLA class II region analysis, no significant differences in CEHs carrying HLA-DRB1*14:54 and DQB1*05:03 were observed between the two groups. We detected only a slight increase in the frequency of the Caucasian European haplotype HLA-A*29:02-B*44:03-C*16:01-DRB1*07:01-DQB1*02:02 in achalasia patients (*pC* = 0.02, OR = 9.32; 95% CI = 1.76–65.52).

**Table 6:** HLA-B/-C/-DRB1/-DQB1 conserved extended haplotypes in achalasia patients and healthy controls.

		Achalasia (N = 182)		Controls (N = 468)	Controls (N = 468)				
	*HLA-B*/*-C*/*-DRB1*/*-DQB1* haplotypes	n	H.F.	Δ'	n	H.F.	Δ'	*pCorr*	OR (95%CI)
**Amerindian**	B*39:05-C*07:02-DRB1*04:07-DQB1*03:02	13	0.0714	0.7129	19	0.0406	0.5025	ns	
	B*35:12-C*04:01-DRB1*08:02-DQB1*04:02	3	0.0164	0.2347	7	0.0150	0.3054	ns	
	B*35:17-C*04:01-DRB1*08:02-DQB1*04:02	3	0.0164	0.3440	14	0.0299	0.7256	ns	
	B*39:06-C*07:02-DRB1*14:06-DQB1*03:01	3	0.0164	0.4670	16	0.0342	0.5482	ns	
	B*40:02-C*03:04-DRB1*16:02-DQB1*03:01	3	0.0164	0.4670	4	0.0086	0.3200	ns	
	B*48:01-C*08:01-DRB1*08:02-DQB1*04:02	3	0.0164	0.5408	8	0.0171	1.0000	ns	
	B*39:05-C*07:02-DRB1*08:02-DQB1*04:02	2	0.0109	-0.0871	5	0.0107	-0.2267	ns	
	B*39:05-C*07:02-DRB1*16:02-DQB1*03:01	2	0.0109	0.0866	3	0.0064	0.0295	ns	
	B*15:15-C*01:02-DRB1*08:02-DQB1*04:02	2	0.0109	1.0000	8	0.0171	0.5251	ns	
	B*39:02-C*07:02-DRB1*16:02-DQB1*03:01	2	0.0109	0.3604	2	0.0043	0.4658	ns	
**Admixed**	B*35:12-C*04:01-DRB1*04:07-DQB1*03:02	5	0.0274	0.4577	2	0.0043	0.0133	ns	
	B*35:01-C*04:01-DRB1*04:07-DQB1*03:02	3	0.0164	0.1459	ND				
	B*40:02-C*03:04-DRB1*04:07-DQB1*03:02	3	0.0164	0.3899	ND				
	B*35:17-C*04:01-DRB1*04:07-DQB1*03:02	2	0.0109	-1.0000	ND				
	B*39:03-C*07:02-DRB1*14:06-DQB1*03:01	2	0.0109	1.0000	ND				
	B*48:01-C*08:01-DRB1*04:04-DQB1*03:02	2	0.0109	0.3809	3	0.0064	0.1472	ns	
	B*35:01-C*07:02-DRB1*04:07-DQB1*03:02	2	0.0109	1.0000	ND				
**Caucasian**	B*44:03-C*16:01-DRB1*07:01-DQB1*02:02	7	0.0384	0.8668	6	0.0128	0.7341	ns	
	B*07:02-C*07:02-DRB1*15:01-DQB1*06:02	3	0.0164	0.5894	7	0.0150	0.4478	ns	
	B*14:02-C*08:02-DRB1*01:02-DQB1*05:01	3	0.0164	0.5850	5	0.0107	0.4414	ns	
	B*14:02-C*08:02-DRB1*11:01-DQB1*03:01	2	0.0109	0.2590	ND				
	B*18:01-C*05:01-DRB1*03:01-DQB1*02:01	2	0.0109	1.0000	3	0.0064	0.5868	ns	
	B*18:01-C*12:03-DRB1*14:54-DQB1*05:03	2	0.0109	0.6447	ND				
	B*38:01-C*12:03-DRB1*14:54-DQB1*05:03	2	0.0109	1.0000	ND				
	B*44:02-C*05:01-DRB1*13:01-DQB1*06:03	2	0.0109	0.4868	1	0.0021	0.2403	ns	
**Caucasian Shared with other populations**	B*41:01-C*17:01-DRB1*13:02-DQB1*06:04	2	0.0109	0.6542	ND		N.D.		
	B*35:01-C*04:01-DRB1*14:54-DQB1*05:03	2	0.0109	0.1473	2	0.0043	0.2252	ns	
	B*52:01-C*12:02-DRB1*15:02-DQB1*06:01	2	0.0109	0.6596	1	0.0021	0.4946	ns	
**African**	B*45:01-C*06:02-DRB1*10:01-DQB1*05:01	2	0.0109	0.6614	ND				
**Unknown**	B*35:03-C*12:03-DRB1*14:54-DQB1*05:03	3	0.0164	0.7335	ND				
	B*15:01-C*03:04-DRB1*04:01-DQB1*03:02	2	0.0109	1.0000	ND				
	B*15:10-C*03:04-DRB1*12:01-DQB1*03:01	2	0.0109	1.0000	ND				
	B*27:05-C*01:02-DRB1*01:01-DQB1*05:01	2	0.0109	1.0000	ND				
	B*51:01-C*14:02-DRB1*04:04-DQB1*03:02	2	0.0109	0.6560	ND				

**H.F.:** Haplotype Frequency; **ns:** not significant; **ND:** Not detected; **∆'**: Delta max; ***p*Corr:** *p* Corrected value using Bonferroni method; **OR:** Odds ratio; **95%CI:** 95% Confidence Interval.

**Table 7:** Most frequent HLA-A/-B/-C/-DRB1/-DQB1 conserved extended haplotypes in Achalasia patients and healthy controls.

		Achalasia (N = 182)				Controls (N = 468)				
	*HLA-A*/*-B*/*-C*/*-DRB1*/*-DQB1* haplotype	n	H.F.	Δ'		n	H.F.	Δ'	*pCorr*	OR (95%CI)
**1**	A*29:02 B*44:03 C*16:01 DRB1*07:01 DQB1*02:02	**7**	**0.0384**	**1.0000**		**2**	**0.0043**	**1.0000**	**0.02º**	**9.32 (1.76–65.52)**
3	A*68:03 B*39:05 C*07:02 DRB1*04:07 DQB1*03:02	5	0.0274	0.4641		5	0.0107	0.2834	ns	
3	A*02:06 B*39:05 C*07:02 DRB1*04:07 DQB1*03:02	3	0.0164	0.1324		5	0.0107	0.1848	ns	
1	A*02:01 B*35:03 C*12:03 DRB1*14:54 DQB1*05:03ª	3	0.0164	1.0000		0	0.0000	N.D		
4	A*02:01 B*15:01 C*03:04 DRB1*04:54 DQB1*03:02	2	0.0109	1.0000		0	0.0000	N.D		
5	A*02:01 B*35:12 C*04:01 DRB1*04:07 DQB1*03:02	2	0.0109	0.1917		1	0.0021	0.3518	ns	
3	A*02:01 B*39:02 C*07:02 DRB1*16:02 DQB1*03:01	2	0.0109	1.0000		0	0.0000	N.D		
5	A*02:01 B*39:05 C*07:02 DRB1*04:07 DQB1*03:02	2	0.0109	-0.4031		6	0.0128	0.1130	ns	
1	A*02:01 B*44:02 C*05:01 DRB1*13:01 DQB1*06:03	2	0.0109	1.0000		0	0.0000	N.D		
3	A*02:06 B*35:17 C*04:01 DRB1*08:02 DQB1*04:02	2	0.0109	0.6240		2	0.0043	0.0517	ns	
5	A*02:06 B*40:02 C*03:04 DRB1*04:07 DQB1*03:02	2	0.0109	0.6240		0	0.0000	N.D		
2	A*11:01 B*35:01 C*04:01 DRB1*14:54 DQB1*05:03ª	2	0.0109	1.0000		0	0.0000	N.D		
3	A*24:02 B*39:06 C*07:02 DRB1*14:06 DQB1*03:01	2	0.0109	0.6174		12	0.0256	0.6992	ns	
2	A*24:02 B*41:01 C*17:01 DRB1*13:02 DQB1*06:04	2	0.0109	1.0000		0	0.0000	N.D		
1	A*25:01 B*18:01 C*12:03 DRB1*14:54 DQB1*05:03ª	2	0.0109	1.0000		0	0.0000	N.D		
6	A*29:02 B*45:01 C*06:02 DRB1*10:01 DQB1*05:01	2	0.0109	1.0000		0	0.0000	N.D		
1	A*33:01 B*14:02 C*08:02 DRB1*01:02 DQB1*05:01	2	0.0109	0.6596		2	0.0043	0.3922	ns	
4	A*33:03 B*51:01 C*14:02 DRB1*04:04 DQB1*03:02	2	0.0109	1.0000		0	0.0000	N.D		
3	A*68:01 B*35:17 C*04:01 DRB1*04:07 DQB1*03:02	2	0.0109	1.0000		0	0.0000	N.D		
5	A*68:01 B*48:01 C*08:01 DRB1*04:04 DQB1*03:02	2	0.0109	1.0000		1	0.0021	0.2761	ns	
4	A*68:02 B*15:10 C*03:04 DRB1*12:01 DQB1*03:01	2	0.0109	1.0000		0	0.0000	N.D		
3	A*68:05 B*39:05 C*07:02 DRB1*04:07 DQB1*03:02	2	0.0109	0.3569		0	0.0000	N.D		

**º**Uncorrected p value: 0.001;

**ª**HLA-DRB1*14:54-DQB1*05:03 carrying haplotypes;

**F.:** Haplotype Frequency; **ns:** not significant; **ND:** Not detected; **∆Not**Delta max; ***pCorr*:** *p* Corrected value using Bonferroni method; **OR:** Odds ratio; **95%CI:** 95% Confidence Interval; **1**: European; **2**: European shared; **3**: Amerindian; **4**: Unknown; **5**: Admixed; **6**: African.

Interestingly, CEHs carrying the susceptibility alleles HLA-DRB1*14:54 and DQB1*05:03, presumably of Eurasian MPA origin, were detected in only achalasia patients and not in the controls (Table 7). No significant associations between these susceptibility alleles and early onset of the disease were found.

## Discussion

Numerous HLA disease case-control studies have demonstrated that an achalasia susceptibility region is associated with the MHC class II genomic transect in the short arm of human six chromosome [5–9,34]. Nevertheless, most studies did not consider the genetic admixtures or probable ancestral origins of the studied populations or the HLA class II alleles and haplotypes associated with achalasia in different populations. In this study, we determined that 1) HLA class I and class II alleles are associated with the susceptibility of Mexican admixed individuals to achalasia using high-resolution sanger sequencing and next-generation HLA typing and 2) the ancestral origin (Native American, European, African and Asian) of these HLA-achalasia-associated alleles and haplotypes. We found a significant association between the HLA class II haplotype HLA-DRB1*14:54-DQB1*05:03 and achalasia, but no significant associations between HLA class I alleles or haplotypes were found. These findings are helpful for understanding that the susceptibility gene(s) are mapped within the HLA class II region and that genetic admixture with Eurasian populations contributes to the presence of these susceptibility loci together with potential environmental triggering factors, such as infections, which results in the development of achalasia.

Specific DNA blocks (with important and predictable patterns of LE) within specific HLA haplotypes are critical for mapping susceptibility or protection alleles in different autoimmune inflammatory conditions [10–11].

Protective effects of the allele HLA-DQB1*02 [6] and the haplotype DRB1*15:01-DQA1*01:02-DQB1*06:02 were reported in Spaniards, whereas DQA1*01:03, DQA1*01:01 and DQB1*06:03 were associated with achalasia in Spaniards [34]. HLA-DQB1*05:02 and DQB1*06:01 have been associated with achalasia susceptibility in Italians [35]. In addition, in 1999, Verne and colleagues described a significant contribution of the DQB1*06:02 allele in the susceptibility of a small group of European American descendants to achalasia [36]. More recent studies in large multinational cohorts of Central European origin support that alleles HLA-DQA1*01:01, HLA-DQB1*05:03 and DQB1*06:01 and their associated haplotypes are strong susceptibility factors to achalasia [8], and their distributions among these populations influence the prevalence of achalasia in Europe. In these studies, the presence of an eight-residue insertion at position 227–234 of the cytoplasmic region of the HLA-DQβ1 chain (specifically encoded in DQB1*05:03 and DQB1*06:01 alleles) determines the susceptibility to achalasia; however, the mechanisms underlying this result are still poorly understood [9].

To our knowledge, this is the first study reporting the distribution of HLA alleles in Mexican mixed-ancestry patients with achalasia using high-resolution typing at the allelic level. We performed admixture estimations that revealed a greater contribution of Native American and European genes and lower contributions of African and Asian genes in patients with achalasia compared to those in healthy controls. Here, we found a significant association between HLA-DQB1*05:03 and HLA-DRB1*14:54 alleles and achalasia, and strong LD results associated the entire HLA-DRB1*14:54-DQB1*0503 haplotype with this clinical condition.

Interestingly, these alleles have been previously associated with pemphigus vulgaris (PV), a rare and severe autoimmune condition, in different populations. Among Europeans, including Slovakians and Serbians, the HLA-DRB1*14:54 and DQB1*05:03 alleles have been strongly associated with PV [37–39]; remarkably, DQB1*05:03 is a strong genetic determinant for PV in non-Jewish groups and Asians [40,41].

Historical reconstructions and HLA association studies with PV support that the DRB1*04:02 and DQB1*03:02 susceptibility genes possibly originated from central Asian populations, including ancient northwest Iranians, and then affected Europe with the migration of Ashkenazi Jews after the 9th century A.D. [42,43]

From this perspective, the presence of the HLA-DRB1*14:54 and DQB1*05:03 alleles in Mexicans could be the result of genetic admixture between Mexicans and Spaniards, who arrived in Mexico in the 16th century. Spaniards came from the western coast of Spain, mainly from the regions of Andalucía, Leon, Extremadura, and the Castillas, as well as from Portugal and Genoa. However, by the end of the colonial period, almost half a million Europeans, mainly peninsular Spaniards but also French, German, and English colonizers, found a place within Mexican society [44]. The *HLA-DRB1* allele has been found at frequencies ranging from 0.0078 to 0.0330 in Irish [45] and Italians [46]. However, HLA-DRB1*14:54 could also reflect admixture events with Asian groups. Asian migrants from South East Asia who arrived throughout the colonial period [47] would have contributed to the presence of this allele in present-day Mexican populations. HLA-DRB1*14:54 has been reported in several East Asian populations, such as Chinese from Hong Kong [48] and Maori and Polynesians from New Zealand [49], in frequencies ranging from 0.0273 to 0.0950. HLA-DQB1*05:03 is present throughout the entire Eurasian region [50], with the highest allelic frequencies being found in Romani from both Spain (0.2672) [51] and the Czech Republic (0.2331) [52]. The Romani people are a traditionally nomadic ethnic group originating in northern India nearly 1500 years ago [53,54].

Moreover, the HLA genes that confer susceptibility to autoimmunity might also be preserved in specific populations because they play a key role in protection against pathogens [55,56]. In Mexican admixed populations, different studies have reported high frequencies of HLA class II alleles, including HLA-DRB1*04:04, DRB1*14:02, and DRB1*01:02 in rheumatoid arthritis (RA); DRB1*03:01 in systemic lupus erythematosus (SLE); and DRB1*11:04 in systemic sclerosis (SSc), that predispose the recipients to different autoimmune disorders [57–59]. Autoimmunity risk HLA class II genes may exhibit increased frequencies due to past selective processes or infectious diseases that developed in different environments, thus partially explaining the susceptibility to autoimmune diseases in our populations [60]. While the pathogenesis of idiopathic achalasia is still unknown, viral, bacterial and neurodegenerative mechanisms have been proposed [3,61–64]. However, the fact that alleles and associations reported elsewhere [5–9] could not be replicated in this study demonstrates the heterogeneity of this disease and that genetic factors in the patient may be linked to environmental factors and the patient’s immune response to both cellular and humoral factors. For instance, all patients with achalasia in previous reports [3,64] were positive for Herpes simplex virus 1 (HSV-1) infection. Since promiscuous binding of the viral sequence to HLA-DR molecules could suggest a potential for HSV-1 to manipulate antigen processing and presentation [62,63], an association among susceptible HLA class II alleles, HVS-1 and a number of triggering factors could detonate an aberrant response that may be linked to an autoimmune origin for achalasia [33, 64]. Furthermore, changes in the binding affinities of HSV-1 viral glycoprotein B and human protein invariant chain (Ii; involved in preventing the premature binding of peptides to clefts in MHC class II molecules) via stabilization of the proline-rich segment of both proteins may indicate the mechanism by which such aberrant responses are achieved, furthering the development of autoimmune conditions (such as achalasia, potentially). Thus, a number of alleles sharing a common sequence of amino acid motifs could potentially be associated with the development of achalasia. Such alleles may have differential distributions in human populations, and ancestry and population genetics studies may aid in their identification.

Idiopathic achalasia may occur at any age and it has been suggested that susceptibility genes inherited to the siblings may influence the age of disease presentation [64]. In our study the age of onset of the disease was heterogeneous and the analysis of distribution of HLA-DRB1/DQB1 susceptibility haplotypes did not display significant associations with early onset of the disease.

It has been also suggested that achalasia may be the result of a self-sustained inflammatory process secondary to acute gastrointestinal infections and that individual susceptibility of developing achalasia following such an initial trigger may be genetically determined. In this complex scenario, neuronal nitric oxide (NO) represents a unique molecule since, depending on its concentration, it is involved in either inhibitory neurotransmission, lower esophageal sphincter relaxation, or defense against infections [65]. A single nucleotide polymorphism has been found within the 3’-untranslated region (UTR) of exon 29. Additionally, an exome analysis of two siblings with infant-onset achalasia revealed homozygosity for a premature stop codon in the gene encoding *NOS1* (at residue Tyr1202, instead of at residue 1435). Kinetic analyses and molecular modeling indicated that the truncated protein product has defective folding capacity, as well as defective capabilities for NO production and binding of co-factors. Other genetic polymorphisms of *NOS* gene isoforms that have been discovered involve the endothelial NOS4a4a, inducible NOS22GA and neuronal NOS29TT [65]. Recently, Sarnelli et al., have provided evidence that genetic variations in the promoter region of the *iNOS* gene are associated with susceptibility to achalasia. Analysis of the allele frequencies revealed that individuals carrying 10 and 13 CCTTT repeats were respectively less and more frequent in achalasia (OR 0.5, 95% CI 0.3–0.5 and OR 1.6, 95% CI 1–2.4, all *p* < 0.05). Long repeats were also significantly associated with an earlier onset of the disease (OR 1.69, 95% CI 1.13–2.53, *p*  =  0.01) [66].

Finally, the high prevalence of ANAs, as well as autoimmune comorbidity in achalasia patients have all favored the hypothesis that achalasia could be an autoimmune disorder [3, 33, 65].

This work has some potential limitations, such as the relatively small sample size, which was limited by the low prevalence of achalasia, and the criterion to recruit only patients born in Mexico whose parents and grandparents were also born in Mexico. With such a modest sample size, we were able to find statistically significant associations and confirm previously reported associations with HLA class II alleles in other populations. Another limitation was the lack of a validation cohort because there are no referral centers in Mexico, and to our knowledge, there is no other cohort of patients with achalasia in Mexico that could potentially validate, or at least replicate, our results.

## Conclusion

MHC class II genes (HLA-DRB1*15:54 and DQB1*05:03) and the conserved haplotype DRB1*14:54-DQB1*05:03 confer risk for the development of achalasia in mixed-ancestry Mexicans. These achalasia-associated MHC class II genes are possibly of Eurasian origin, and they could be important in the development of aberrant immune responses against triggering factors, such as viral, bacterial or parasitic infections, that may lead to the development of this autoimmune condition.

## Supporting information

S1 Table Gene frequencies of HLA-A in achalasia patients and healthy controls. (DOCX)

S2 Table Gene frequencies of HLA-B in achalasia patients and healthy controls. (DOCX)

S3 Table Gene frequencies of HLA-C in achalasia patients and healthy controls. (DOCX)

S4 Table Frequencies of HLA-B/-C block in achalasia patients and healthy controls. (DOCX)

S1 Data Data_Raw data HLA, arlequin analysis, haplotypes, LD, Mexican healthy controls. (XLSX)

## Abbreviations

ANAs: antinuclear antibodies
CEH: conserved extended haplotype
CI: confidence interval
EH: expected heterozygosity
EM: expectation-maximization
GF: gene frequency
GSSP: group-specific sequencing primer
HF: haplotype frequency
HLA: human leukocyte antigen
HSV-1: herpes simplex virus 1
HWE: Hardy-Weinberg equilibrium
LD: linkage disequilibrium
LES: lower esophageal sphincter
MHC: major histocompatibility complex
MPA: most probable ancestry
OH: observed heterozygosity
OR: odds ratio
PBMC: peripheral blood mononuclear cells
pC: p-corrected value
PCR: polymerase chain reaction
PD: power of discrimination
PIC: polymorphism information content
PV: pemphigus vulgaris
RA: rheumatoid arthritis
SBM: sequence-based method
SLE: systemic lupus erythematosus
SSc: systemic sclerosis
STR: short tandem repeats
Δ: delta value
Δ’: relative delta value
