# Supplementary material for: An original Eurasian haplotype, HLA-DRB1*14:54-DQB1*05:03, influences the susceptibility to idiopathic achalasia
Source: PLoS One. 2018 Aug 9;13(8):e0201676. doi: 10.1371/journal.pone.0201676 (PMC6084941; doi:10.1371/journal.pone.0201676)
Supplement: S1 Table — (DOCX) [file pone.0201676.s001.docx]

**Supplementary Table 1. Gene frequencies of HLA-A in Achalasia patients and healthy controls.**

|  | **Achalasia (N = 182)** | | **Controls (N = 468)** | |  |  |
| --- | --- | --- | --- | --- | --- | --- |
| **Allele** | **n** | **G.F.** | **n** | **G.F.** | ***p*Corr** | **OR (95%CI)** |
| A*01:01 | 11 | 0.0604 | 17 | 0.0363 | ns |  |
| A*02:01 | 46 | 0.2527 | 107 | 0.2286 | ns |  |
| A*02:05 | 3 | 0.0164 | 8 | 0.0171 | ns |  |
| A*02:06 | 20 | 0.1099 | 45 | 0.0962 | ns |  |
| A*02:58 | 1 | 0.0054 | ND |  |  |  |
| A*02:398 | 1 | 0.0054 | ND |  |  |  |
| A*03:01 | 6 | 0.0330 | 15 | 0.0321 | ns |  |
| A*03:02 | 1 | 0.0054 | 3 | 0.0064 | ns |  |
| A*11:01 | 3 | 0.0164 | 10 | 0.0214 | ns |  |
| A*11:JZDV | 1 | 0.0054 | ND |  |  |  |
| A*23:01 | 2 | 0.0110 | 8 | 0.0171 | ns |  |
| A*24:02 | 25 | 0.1374 | 79 | 0.1688 | ns |  |
| A*24:25 | 1 | 0.0054 | 3 | 0.0064 | ns |  |
| A*25:01 | 2 | 0.0110 | 3 | 0.0064 | ns |  |
| A*26:01 | 1 | 0.0054 | 9 | 0.0192 | ns |  |
| A*29:01 | 1 | 0.0054 | ND |  |  |  |
| A*29:02 | 10 | 0.0549 | 12 | 0.0256 | ns |  |
| A*30:02 | 3 | 0.0164 | 7 | 0.0150 | ns |  |
| A*31:01 | 7 | 0.0385 | 37 | 0.0791 | ns |  |
| A*32:01 | 2 | 0.0110 | 4 | 0.0085 | ns |  |
| A*33:01 | 4 | 0.0219 | 6 | 0.0128 | ns |  |
| A*33:03 | 2 | 0.0110 | 2 | 0.0043 | ns |  |
| A*66:01 | 1 | 0.0054 | 6 | 0.0128 | ns |  |
| A*66:02 | 1 | 0.0054 | ND |  |  |  |
| A*68:01 | 9 | 0.0495 | 37 | 0.0791 | ns |  |
| A*68:02 | 2 | 0.0109 | 14 | 0.0299 | ns |  |
| A*68:03 | 10 | 0.0549 | 16 | 0.0342 | ns |  |
| A*68:05 | 5 | 0.0275 | 3 | 0.0064 | ns |  |
| A*69:01 | 1 | 0.0054 | ND |  |  |  |
