# Supplementary material for: An original Eurasian haplotype, HLA-DRB1*14:54-DQB1*05:03, influences the susceptibility to idiopathic achalasia
Source: PLoS One. 2018 Aug 9;13(8):e0201676. doi: 10.1371/journal.pone.0201676 (PMC6084941; doi:10.1371/journal.pone.0201676)
Supplement: S2 Table — (DOCX) [file pone.0201676.s002.docx]

**Supplementary Table 2. Gene frequencies of HLA-B in Achalasia patients and healthy controls.**

|  | **Achalasia (N = 182)** | | **Controls (N = 468)** | |  |  |
| --- | --- | --- | --- | --- | --- | --- |
| **Allele** | **n** | **G.F.** | **n** | **G.F.** | ***pCorr*** | **OR (95%CI)** |
| B*07:02 | 5 | 0.0274 | 19 | 0.0406 | ns |  |
| B*07:05 | 1 | 0.0054 | ND |  |  |  |
| B*08:01 | 3 | 0.0164 | 3 | 0.0064 | ns |  |
| B*13:02 | 1 | 0.0054 | 6 | 0.0128 | ns |  |
| B*14:01 | 1 | 0.0054 | 4 | 0.0085 | ns |  |
| B*14:02 | 7 | 0.0385 | 15 | 0.0321 | ns |  |
| B*15:01 | 5 | 0.0275 | 10 | 0.0214 | ns |  |
| B*15:03 | 1 | 0.0054 | 2 | 0.0043 | ns |  |
| B*15:10 | 2 | 0.0109 | 1 | 0.0021 | ns |  |
| B*15:15 | 2 | 0.0109 | 15 | 0.0321 | ns |  |
| B*15:17 | 1 | 0.0054 | 3 | 0.0064 | ns |  |
| B*15:30 | 2 | 0.0109 | 8 | 0.0171 | ns |  |
| B*18:01 | 6 | 0.0330 | 8 | 0.0171 | ns |  |
| B*27:03 | 1 | 0.0054 | 1 | 0.0021 | ns |  |
| B*27:05 | 2 | 0.0109 | 3 | 0.0064 | ns |  |
| B*35:01 | 10 | 0.0549 | 27 | 0.0577 | ns |  |
| B*35:02 | 2 | 0.0110 | 2 | 0.0043 | ns |  |
| B*35:03 | 4 | 0.0219 | 5 | 0.0107 | ns |  |
| B*35:05 | 1 | 0.0054 | ND |  |  |  |
| B*35:08 | 1 | 0.0054 | 3 | 0.0064 | ns |  |
| B*35:09 | 1 | 0.0054 | ND |  |  |  |
| B*35:12 | 9 | 0.0495 | 18 | 0.0385 | ns |  |
| B*35:17 | 6 | 0.0330 | 18 | 0.0385 | ns |  |
| B*35:43 | 1 | 0.0054 | 9 | 0.0192 | ns |  |
| B*37:01 | 1 | 0.0054 | 4 | 0.0085 | ns |  |
| B*38:01 | 4 | 0.0219 | 6 | 0.0128 | ns |  |
| B*39:01 | 2 | 0.0109 | 5 | 0.0107 | ns |  |
| B*39:02 | 5 | 0.0275 | 10 | 0.0214 | ns |  |
| B*39:03 | 2 | 0.0109 | ND |  |  |  |
| B*39:05 | 18 | 0.0989 | 37 | 0.0791 | ns |  |
| B*39:06 | 7 | 0.0385 | 32 | 0.0684 | ns |  |
| B*39:08 | 2 | 0.0109 | 3 | 0.0064 | ns |  |
| B*40:01 | 1 | 0.0054 | ND |  |  |  |
| B*40:02 | 9 | 0.0495 | 25 | 0.0534 | ns |  |
| B*40:06 | 2 | 0.0109 | ND |  |  |  |
| B*40:27 | 1 | 0.0054 | 4 | 0.0085 | ns |  |
| B*41:01 | 6 | 0.0330 | 5 | 0.0107 | ns |  |
| B*41:02 | 1 | 0.0054 | ND |  |  |  |
| B*44:02 | 4 | 0.0219 | 5 | 0.0107 | ns |  |
| B*44:03 | 9 | 0.0495 | 13 | 0.0278 | ns |  |
| B*45:01 | 4 | 0.0219 | 3 | 0.0064 | ns |  |
| B*48:01 | 7 | 0.0385 | 20 | 0.0427 | ns |  |
| B*48:03 | 1 | 0.0054 | ND |  |  |  |
| B*49:01 | 2 | 0.0109 | 9 | 0.0192 | ns |  |
| B*50:01 | 1 | 0.0054 | 4 | 0.0085 | ns |  |
| B*51:01 | 6 | 0.0329 | 28 | 0.0598 | ns |  |
| B*52:01 | 4 | 0.0219 | 10 | 0.0214 | ns |  |
| B*55:01 | 2 | 0.0109 | 3 | 0.0064 | ns |  |
| B*57:01 | 2 | 0.0109 | 7 | 0.0150 | ns |  |
| B*58:01 | 3 | 0.0164 | 3 | 0.0064 | ns |  |
| B*78:01 | 1 | 0.0054 | ND |  |  |  |
