# Supplementary material for: An original Eurasian haplotype, HLA-DRB1*14:54-DQB1*05:03, influences the susceptibility to idiopathic achalasia
Source: PLoS One. 2018 Aug 9;13(8):e0201676. doi: 10.1371/journal.pone.0201676 (PMC6084941; doi:10.1371/journal.pone.0201676)
Supplement: S3 Table — (DOCX) [file pone.0201676.s003.docx]

**Supplementary Table 3. Gene frequencies of HLA-C in Achalasia patients and healthy controls.**

|  | **Achalasia (N = 182)** | | | **Controls (N = 468)** | |  |  |
| --- | --- | --- | --- | --- | --- | --- | --- |
| **Allele** | | **n** | **G.F.** | **n** | **G.F.** | ***pCorr*** | **OR (95%CI)** |
| C*01:02 | | 10 | 0.0549 | 42 | 0.0897 | ns |  |
| C*02:02 | | 1 | 0.0054 | 6 | 0.0128 | ns |  |
| C*02:10 | | 1 | 0.0054 | 2 | 0.0043 | ns |  |
| C*03:02 | | 1 | 0.0054 | 2 | 0.0043 | ns |  |
| C*03:03 | | 3 | 0.0164 | 14 | 0.0299 | ns |  |
| C*03:04 | | 11 | 0.0604 | 31 | 0.0662 | ns |  |
| C*03:05 | | 4 | 0.0219 | 16 | 0.0342 | ns |  |
| C*04:01 | | 31 | 0.1703 | 87 | 0.1859 | ns |  |
| C*05:01 | | 6 | 0.0330 | 10 | 0.0214 | ns |  |
| C*05:09 | | 1 | 0.0054 | ND |  |  |  |
| C*06:02 | | 7 | 0.0385 | 28 | 0.0598 | ns |  |
| C*07:01 | | 10 | 0.0549 | 25 | 0.0534 | ns |  |
| C*07:02 | | 40 | 0.2198 | 97 | 0.2073 | ns |  |
| C*07:18 | | 2 | 0.0109 | ND |  |  |  |
| C*08:01 | | 7 | 0.0385 | 22 | 0.0470 | ns |  |
| C*08:02 | | 8 | 0.0440 | 19 | 0.0406 | ns |  |
| C*08:03 | | 2 | 0.0109 | 4 | 0.0085 | ns |  |
| C*12:02 | | 3 | 0.0164 | 2 | 0.0043 | ns |  |
| C*12:03 | | 12 | 0.0659 | 12 | 0.0256 | ns |  |
| C*14:02 | | 3 | 0.0165 | 4 | 0.0085 | ns |  |
| C*15:02 | | 4 | 0.0219 | 9 | 0.0192 | ns |  |
| C*15:05 | | 1 | 0.0054 | 1 | 0.0021 | ns |  |
| C*16:01 | | 10 | 0.0549 | 12 | 0.0256 | ns |  |
| C*17:01 | | 4 | 0.0219 | 1 | 0.0021 | ns |  |

**G.F.:** Gene Frequency; **ns:** not significant; ND: Not detected; ***pCorr:*** *p* Corrected value using Bonferroni method; **OR:** Odds ratio; **95%CI:** 95 % Confidence Interval.
