# Supplementary material for: An original Eurasian haplotype, HLA-DRB1*14:54-DQB1*05:03, influences the susceptibility to idiopathic achalasia
Source: PLoS One. 2018 Aug 9;13(8):e0201676. doi: 10.1371/journal.pone.0201676 (PMC6084941; doi:10.1371/journal.pone.0201676)
Supplement: S4 Table — (DOCX) [file pone.0201676.s004.docx]

**Supplementary Table 4. Frequencies of HLA-B/-C block in Achalasia patients and healthy controls**

|  |  | **Achalasia (N = 182)** | | | **Controls (N = 468)** | | |  |  |
| --- | --- | --- | --- | --- | --- | --- | --- | --- | --- |
|  | ***HLA-B*/*-C* haplotypes** | **n** | **H.F.** | **Δ'** | **n** | **H.F.** | **Δ'** | ***pCorr*** | **OR (95% CI)** |
| **Amerindian** | B*39:05-C*07:02 | 18 | 0.0989 | 0.9288 | 34 | 0.0726 | 0.8975 | ns |  |
|  | B*35:12-C*04:01 | 10 | 0.0549 | 1.0000 | 16 | 0.0341 | 0.8632 | ns |  |
|  | B*35:17-C*04:01 | 6 | 0.0330 | 0.7991 | 17 | 0.0363 | 1.0000 | ns |  |
|  | B*39:06-C*07:02 | 6 | 0.0330 | 0.8169 | 27 | 0.0576 | 0.8025 | ns |  |
|  | B*40:02-C*03:04 | 6 | 0.0330 | 0.6452 | 11 | 0.0235 | 0.4196 | ns |  |
|  | B*39:02-C*07:02 | 5 | 0.0275 | 1.0000 | 4 | 0.0086 | 0.2414 | ns |  |
|  | B*48:01-C*08:01 | 5 | 0.0275 | 0.7029 | 15 | 0.0320 | 0.7376 | ns |  |
|  | B*15:01-C*01:02 | 2 | 0.0109 | 0.3651 | 7 | 0.0149 | 0.6701 | ns |  |
|  | B*15:30-C*01:02 | 2 | 0.0109 | 1.0000 | 8 | 0.0170 | 1.0000 | ns |  |
|  | B*35:01-C*07:02 | 2 | 0.0109 | -0.0900 | 1 | 0.0021 | -0.3126 | ns |  |
|  | B*39:08-C*07:02 | 2 | 0.0109 | 1.0000 | 3 | 0.0064 | 1.0000 | ns |  |
|  | B*40:02-C*03:05 | 2 | 0.0109 | 0.4740 | 10 | 0.0213 | 0.6045 | ns |  |
|  | B*48:01-C*08:03 | 2 | 0.0109 | 1.0000 | 3 | 0.0064 | 0.7387 | ns |  |
|  | B*15:17-C*07:01 | 1 | 0.0054 | 1.0000 | 3 | 0.0064 | 1.0000 | ns |  |
|  | B*39:01-C*07:02 | 1 | 0.0054 | 0.3701 | 4 | 0.0085 | 0.7471 | ns |  |
|  | B*39:06-C*03:05 | 1 | 0.0054 | 0.2219 | 1 | 0.0021 | -0.0938 | ns |  |
| **European** | **B*44:03-C*16:01** | **9** | **0.0494** | **0.8824** | **8** | **0.0170** | **0.6571** | **0.0200** | **2.9 (1.04-8.66)** |
|  | B*07:02-C*07:02 | 5 | 0.0275 | 1.0000 | 15 | 0.0320 | 0.7893 | ns |  |
|  | B*38:01-C*12:03 | 5 | 0.0275 | 1.0000 | 6 | 0.0128 | 1.0000 | ns |  |
|  | B*44:02-C*05:01 | 4 | 0.0219 | 0.7925 | 4 | 0.0086 | 0.7956 | ns |  |
|  | B*18:01-C*12:03 | 3 | 0.0164 | 0.4647 | 2 | 0.0043 | 0.2301 | ns |  |
|  | B*41:01-C*17:01 | 3 | 0.0164 | 0.7415 | 1 | 0.0021 | 1.0000 | ns |  |
|  | B*08:01-C*07:01 | 3 | 0.0164 | 1.0000 | 3 | 0.0064 | 1.0000 | ns |  |
|  | B*15:01-C*03:04 | 2 | 0.0109 | 0.3614 | 2 | 0.0043 | 0.1427 | ns |  |
|  | B*18:01-C*05:01 | 2 | 0.0109 | 0.3106 | 5 | 0.0107 | 0.6167 | ns |  |
|  | B*18:01-C*07:01 | 1 | 0.0054 | 0.1213 | ND |  |  |  |  |
|  | B*37:01-C*06:02 | 1 | 0.0054 | 1.0000 | 3 | 0.0064 | 0.7339 | ns |  |
|  | B*44:03-C*04:01 | 1 | 0.0054 | -0.4174 | 3 | 0.0064 | 0.0533 | ns |  |
|  | B*50:01-C*06:02 | 1 | 0.0054 | 1.0000 | 4 | 0.0085 | 1.0000 | ns |  |
|  | B*57:01-C*06:02 | 1 | 0.0054 | 0.4812 | 2 | 0.0042 | 0.2398 | ns |  |
|  | B*57:01-C*07:01 | 1 | 0.0054 | 0.4728 | ND |  |  |  |  |
| **European Shared with other populations** | B*35:01-C*04:01 | 8 | 0.0440 | 0.7589 | 15 | 0.0320 | 0.4530 | ns |  |
|  | B*14:02-C*08:02 | 7 | 0.0385 | 1.0000 | 11 | 0.0235 | 0.7219 | ns |  |
|  | B*51:01-C*15:02 | 3 | 0.0164 | 0.7415 | 9 | 0.0192 | 1.0000 | ns |  |
|  | B*52:01-C*12:02 | 3 | 0.0164 | 1.0000 | 2 | 0.0043 | 1.0000 | ns |  |
|  | B*41:01-C*07:01 | 2 | 0.0109 | 0.2971 | 4 | 0.0086 | 0.7886 | ns |  |
|  | B*13:02-C*06:02 | 1 | 0.0054 | 1.0000 | 5 | 0.0106 | 0.8226 | ns |  |
|  | B*14:01-C*08:02 | 1 | 0.0054 | 1.0000 | 4 | 0.0085 | 1.0000 | ns |  |
| **Asian** | B*35:03-C*12:03 | 4 | 0.0219 | 1.0000 | 1 | 0.0021 | 0.1788 | ns |  |
|  | B*51:01-C*14:02 | 3 | 0.0164 | 1.0000 | 3 | 0.0064 | 0.7339 | ns |  |
|  | B*58:01-C*03:02 | 1 | 0.0054 | 0.4894 | 1 | 0.0021 | 0.4967 | ns |  |
| **African** | B*45:01-C*06:02 | 3 | 0.0164 | 0.7400 | 2 | 0.0043 | 0.6453 | ns |  |
|  | B*49:01-C*07:01 | 2 | 0.0109 | 1.0000 | 6 | 0.0128 | 0.6477 | ns |  |
|  | B*58:01-C*07:18 | 2 | 0.0109 | 1.0000 | ND |  |  |  |  |
|  | B*15:03-C*02:10 | 1 | 0.0054 | 1.0000 | 1 | 0.0021 | 0.4978 | ns |  |
|  | B*45:01-C*16:01 | 1 | 0.0054 | 0.2092 | 1 | 0.0021 | 0.3156 | ns |  |
| **Unknown** | B*15:10-C*03:04 | 2 | 0.0109 | 1.0000 | ND |  |  |  |  |
|  | B*15:15-C*01:02 | 2 | 0.0109 | 0.6466 | 13 | 0.0277 | 0.8534 | ns |  |
|  | B*27:05-C*01:02 | 2 | 0.0109 | 1.0000 | 1 | 0.0021 | 0.2670 | ns |  |
|  | B*35:02-C*04:01 | 2 | 0.0109 | 1.0000 | ND |  |  |  |  |
|  | B*35:08-C*04:01 | 2 | 0.0109 | 1.0000 | 3 | 0.0064 | 1.0000 | ns |  |
|  | B*39:03-C*07:02 | 2 | 0.0109 | 1.0000 | ND |  |  |  |  |
|  | B*07:05-C*15:05 | 1 | 0.0054 | 1.0000 | ND |  |  |  |  |
|  | B*15:01-C*03:03 | 1 | 0.0054 | 0.3157 | 1 | 0.0021 | 0.0721 | ns |  |
|  | B*15:15-C*03:03 | 1 | 0.0054 | 0.3228 | ND |  |  |  |  |
|  | B*27:03-C*05:01 | 1 | 0.0054 | 1.0000 | ND |  |  |  |  |
|  | B*35:05-C*12:03 | 1 | 0.0054 | 1.0000 | ND |  |  |  |  |
|  | B*35:09-C*04:01 | 1 | 0.0054 | 1.0000 | ND |  |  |  |  |
|  | B*35:14-C*04:01 | 1 | 0.0054 | 1.0000 | 6 | 0.0128 | 1.0000 | ns |  |
|  | B*35:17-C*03:04 | 1 | 0.0054 | 0.0724 | ND |  |  |  |  |
|  | B*35:43-C*01:02 | 1 | 0.0054 | 1.0000 | ND |  |  |  |  |
|  | B*39:01-C*04:01 | 1 | 0.0054 | 0.3821 | ND |  |  |  |  |
|  | B*39:05-C*01:02 | 1 | 0.0054 | -0.0202 | 1 | 0.0021 | -0.7014 | ns |  |
|  | B*40:01-C*04:01 | 1 | 0.0054 | 1.0000 | ND |  |  |  |  |
|  | B*40:06-C*03:05 | 1 | 0.0054 | 0.4894 | ND |  |  |  |  |
|  | B*40:06-C*15:02 | 1 | 0.0054 | 0.4894 | ND |  |  |  |  |
|  | B*40:27-C*08:01 | 1 | 0.0054 | 1.0000 | 1 | 0.0021 | 0.2127 | ns |  |
|  | B*41:01-C*04:01 | 1 | 0.0054 | -0.1261 | ND |  |  |  |  |
|  | B*41:02-C*17:01 | 1 | 0.0054 | 1.0000 | ND |  |  |  |  |
|  | B*44:02-C*05:09 | 1 | 0.0054 | 1.0000 | ND |  |  |  |  |
|  | B*48:03-C*08:01 | 1 | 0.0054 | 1.0000 | ND |  |  |  |  |
|  | B*51:01-C*02:02 | 1 | 0.0054 | 0.4812 | ND |  |  |  |  |
|  | B*52:01-C*03:02 | 1 | 0.0054 | 0.4867 | ND |  |  |  |  |
|  | B*52:01-C*04:01 | 1 | 0.0054 | 0.0114 | ND |  |  |  |  |
|  | B*55:01-C*03:03 | 1 | 0.0054 | 0.4921 | ND |  |  |  |  |
|  | B*58:01-C*12:02 | 1 | 0.0054 | 0.2342 | ND |  |  |  |  |
|  | B*78:01-C*16:01 | 1 | 0.0054 | 1.0000 | ND |  |  |  |  |

**H.F.:** Haplotype Frequency; **ns:** not significant; **ND:** Not detected; **∆':** Delta max; ***pCorr:*** *p* Corrected value using Bonferroni method; **OR:** Odds ratio; **95%CI:** 95 % Confidence Interval.
